# Effects of Dimethyl Fumarate on Brain Atrophy in Relapsing-Remitting Multiple Sclerosis: Pooled Analysis Phase 3 DEFINE and CONFIRM Studies

**DOI:** 10.3389/fneur.2022.809273

**Published:** 2022-03-18

**Authors:** Kunio Nakamura, Oksana Mokliatchouk, Douglas L. Arnold, Tarek A. Yousry, Ludwig Kappos, Nancy Richert, Katherine Ayling-Rouse, Catherine Miller, Elizabeth Fisher

**Affiliations:** ^1^Department of Biomedical Engineering, Lerner Research Institute, Cleveland Clinic, Cleveland, OH, United States; ^2^Biogen, Cambridge, MA, United States; ^3^McConnell Brain Imaging Center, Montreal Neurological Institute, McGill University, Montreal, QC, Canada; ^4^Lysholm Department of Neuroradiology, National Hospital for Neurology and Neurosurgery, University College London Institute of Neurology, London, United Kingdom; ^5^Research Center for Clinical Neuroimmunology and Neuroscience Basel (RC2NB), Departments of Medicine, Clinical Research, Biomedicine and Biomedical Engineering, University Hospital and University of Basel, Basel, Switzerland; ^6^Envision Pharma Group, Horsham, United Kingdom

**Keywords:** dimethyl fumarate, multiple sclerosis, brain parenchymal fraction, pseudoatrophy, brain volume loss

## Abstract

**Objective:**

In the pivotal DEFINE and CONFIRM trials for dimethyl fumarate (DMF), patterns of brain volume changes were different, potentially due to low sample sizes and because MRIs were analyzed at two different reading centers. We evaluated effects of DMF on brain volume change in patients with multiple sclerosis (MS) through reanalysis of pooled images from DEFINE/CONFIRM trials in one reading center.

**Methods:**

MRIs from DEFINE/CONFIRM at weeks 0, 24, 48, and 96 from patients randomized to twice-daily DMF or placebo (PBO) were reanalyzed at the Cleveland Clinic to measure brain parenchymal fraction (BPF). To account for pseudoatrophy, brain volume estimates were re-baselined to calculate changes for weeks 48–96.

**Results:**

Across studies, 301 and 314 patients receiving DMF and PBO, respectively, had analyzable MRIs. In weeks 0–48, mean ± SE percentage change in BPF was −0.44 ± 0.04 vs. −0.34 ± 0.04% in DMF vs. PBO, respectively, whereas in weeks 48–96, mean ± SE percentage change in BPF was −0.27 ± 0.03 vs. −0.41 ± 0.04% in DMF vs. PBO, respectively. The mixed-effect model for repeated measures showed similar results: in weeks 48–96, estimated change (95% confidence interval) in BPF was −0.0021 (−0.0027, −0.0016) for DMF vs. −0.0033 (−0.0039, −0.0028) for PBO (35.9% reduction; *p* = 0.0025).

**Conclusions:**

The lower rate of whole brain volume loss with DMF in this pooled BPF analysis in the second year vs. PBO is consistent with its effects on relapses, disability, and MRI lesions. Brain volume changes in the first year may be explained by pseudoatrophy effects also described in other MS clinical trials.

## Introduction

Brain tissue loss occurs as a consequence of multiple sclerosis (MS) disease processes such as demyelination, axonal loss, and neurodegeneration. It can be detected from the earliest stages of MS and throughout the disease course (1) using analysis of serial MRI. Brain volume loss progresses at a faster rate in patients with MS than in healthy individuals (2), and it correlates with disability progression and cognitive impairment (3, 4). Quantification of brain volume loss from MRI is considered a marker of MS destructive pathology and is commonly used in clinical trials to assess treatment response.

Several controlled clinical trials of approved MS disease-modifying therapies have demonstrated a treatment effect on brain atrophy, with reductions in brain volume loss ranging from 20 to 50% (5, 6). However, brain volume changes due to MS are small (typically, <1% per year) (7), and the measurements are technique- and center-dependent (8–11), and volume changes unrelated to tissue loss may confound results. For example, controlled studies of high-dose corticosteroids, as well as several disease-modifying MS therapies, such as interferon (IFN)-β, natalizumab, and cladribine, have shown accelerated brain volume loss in the first several months after treatment initiation (12–14). This early effect is termed “pseudoatrophy” and is presumed to be due to fluid shifts rather than true tissue loss (15, 16).

Delayed-release dimethyl fumarate (DMF) is an oral treatment approved for adult patients with relapsing-remitting MS (RRMS). In the pivotal phase 3, randomized, placebo (PBO)-controlled DEFINE and CONFIRM trials, DMF significantly reduced the annualized relapse rate and risk of MS relapse compared with PBO (17, 18). Furthermore, in the MRI cohort, which comprised 44 and 48% of the full study population of DEFINE and CONFIRM, respectively, significant reductions in the number of new and enlarging T2 lesions and gadolinium-enhancing (Gd+) lesions were also observed. Changes in brain volume were assessed in both phase 3 studies using SIENA (19) to calculate percentage brain volume change (PBVC). In each study, after the initial study period, there was a significant reduction in the rate of brain atrophy (30.3% reduction from 6 months to 2 years in DEFINE; 32.2% reduction from year 1 to year 2 in CONFIRM) in the DMF twice-daily (BID) arm compared with PBO (20, 21). However, MRI analyses for the two studies were performed at two different central reading centers, and different reference time points were used for the brain atrophy outcomes (19). PBVC measurements are dependent on how SIENA is implemented at a given MRI reading center (22). As such, the original PBVC measurements in DEFINE and CONFIRM were analyzed separately and brain atrophy data were not included in the previously reported pre-planned *post hoc* analysis of the integrated DEFINE and CONFIRM datasets (23).

The objective of this study was to pool the MRIs from DEFINE and CONFIRM and reanalyze the images at a single, independent MRI analysis center to clarify the effects of DMF BID compared with PBO on whole brain atrophy over 2 years in a large group of patients with relapsing MS.

## Materials and Methods

### Study Design

The designs of the pivotal phase 3 DEFINE (NCT00420212) and CONFIRM (NCT00451451) studies have been described elsewhere; overall design, inclusion/exclusion criteria, and efficacy and safety measurement criteria were similar between the studies (17, 18). Per protocol, natalizumab was discontinued within the 6 months prior to randomization. In addition, for inclusion, patients may not have received prior treatment with IFN-α, IFN-β, or glatiramer acetate within the 3 months prior to randomization. Patients were randomized to receive DMF 240 mg twice or thrice daily, PBO, or glatiramer acetate for up to 96 weeks. For this analysis, data from patients participating in DEFINE and CONFIRM were pooled, and they were included in this reanalysis if the patients (1) were part of the MRI cohort in either DEFINE or CONFIRM and (2) had been randomized to either the DMF BID or PBO arm.

Rules for switching to rescue therapy varied slightly between studies. Patients were eligible to switch to an alternative MS therapy if they (1) had completed 48 weeks of study treatment and had at least one (DEFINE) or two (CONFIRM) confirmed relapses after 24 weeks; or (2) had confirmed disability progression at any time in either DEFINE or CONFIRM.

### MRI Imaging and Brain Atrophy Assessments

MRI scans were obtained at baseline and at weeks 24, 48, and 96 in patients from a subset of sites with full MRI capabilities, as previously described (20, 21). The same MRI acquisition protocol was followed for both studies, with the exception of the proton density (PD)-weighted and T2-weighted images, which were acquired as a dual echo scan in CONFIRM and as separate acquisitions in DEFINE. MRIs were not performed within 30 days following a course of steroids.

MRI scans were transferred from the original trials' MRI reading centers (NeuroRx Research, Montreal, Canada [DEFINE] and UCL Queen Square Institute of Neurology, London, United Kingdom [CONFIRM]) to the Cleveland Clinic MS MRI Analysis Center (Cleveland, OH, United States) for reanalysis. Following pre-processing to reduce noise and non-uniform intensity variations (24, 25), images were processed and analyzed to calculate brain parenchymal fraction (BPF) at each time point. PD- and T2-weighted images were co-registered, if necessary, and BPF was assessed from PD- and T2-weighted images using an automated technique developed in-house (autosegMS, Cleveland Clinic). With this algorithm, BPF was defined as the brain volume divided by the volume of the outer contour of the brain (an estimate of total intracranial volume) (14, 26). Images and segmentation results were visually verified for quality control by trained personnel blinded to treatment assignment.

### Primary Analysis

The percentage change in BPF from baseline to week 48 and from week 48 to week 96 was calculated in patients who had BPF measurements at the corresponding time points. The percentage change in BPF was calculated at each follow-up time point as %dBPF = (BPF_tpt_ – BPF_reference_) / BPF_reference_ × 100%, with BPF_reference_ defined as baseline, for the change in BPF from baseline to week 48; and week 48, for the change in BPF from week 48 to week 96.

A repeated measures model was used to assess the change from baseline in BPF in patients with a baseline BPF measurement and more than one post-baseline BPF measurement. The mixed-effect model for repeated measures was considered most appropriate, as it used data at all time points. The model included treatment, study, week, and their two-way and three-way interactions and was adjusted for region and BPF at baseline; an unstructured variance-covariance structure was used. Due to decline in BPF in the first year as a result of pseudoatrophy, brain volume estimates were re-baselined to calculate changes from week 48 to week 96, and therefore the hypotheses tested were the null hypotheses of no difference between treatment groups with respect to change in weeks 48–96. Adjusted mean differences (with 95% confidence intervals [CIs]) between treatment groups (DMF BID vs. PBO) for the change in BPF between week 48 to week 96 are also reported.

### Additional Analyses

A sensitivity analysis was conducted in which the primary analysis was repeated, excluding data collected after patients switched to an alternative MS medication (for 22 and 49 patients receiving DMF and PBO, respectively). Measurements collected before the switch for these patients were included in the analyses (20 and 46 patients receiving DMF and PBO, respectively, had data before the switch).

Correlations between percentage change in BPF and the original PBVC measurements were calculated using Spearman's correlation coefficient for each study separately. Mean brain volume changes were compared between subgroups of patients with and without Gd+ lesions at baseline. Associations between brain volume changes and disability progression were assessed for both the original PBVC measurements and the percentage change in BPF by comparing brain volume changes in subgroups of patients with confirmed disability progression vs. stable patients.

### Standard Protocol Approvals, Registrations, and Patient Consents

Studies were approved by the relevant Institutional Review Board for each study site, and each study was conducted in accordance with the Declaration of Helsinki, International Conference on Harmonization Good Clinical Practice guidelines, and all applicable laws and regulations. All participants provided written informed consent before study procedures.

### Data Availability

DEFINE and CONFIRM were registered with ClinicalTrials.gov (NCT00420212 and NCT00451451). Requests for data supporting this article should be submitted to the Biogen Clinical Data Request Portal (biogenclinicaldatarequest.com).

## Results

### Patient Baseline Characteristics

Demographic and clinical characteristics of patients included in the DEFINE (*N* = 1,234) and CONFIRM (*N* = 1,417) intention-to-treat populations have been reported previously (17, 18). A total of 1,221 patients were included in the MRI cohorts; of those, 615 (50%) were randomized to DMF BID or PBO and were included in the pooled atrophy analysis. There were no significant differences in disease characteristics between the DMF BID group and the PBO group at baseline (Table 1).

**Table 1:** Key baseline demographics and disease characteristics.

**Characteristic**	**PBO** ***N* = 314**	**DMF BID** ***N* = 301**
Age, mean ± SD, years	37.5 ± 9.1	38.1 ± 9.0
Female, n (%)	228 (73)	217 (72)
McDonald criteria, n (%)		
1 criterion	268 (85)	248 (82)
2–4 criteria	46 (15)	53 (18)
Time since first MS symptoms, mean (range), years	8.2 (0–32)	8.3 (0–42)
MS relapses in previous year, mean ± SD	1.3 ± 0.7	1.3 ± 0.7
Prior approved MS treatment, n (%)	159 (51)	145 (48)
Baseline EDSS score, mean ± SD	2.5 ± 1.2	2.4 ± 1.2
T2 lesion volume, mean ± SD, mm^3^	9,957.6 ± 11,280.3^a^	10,785.6 ± 11,398.0
T1-hypointense lesion volume, mean ± SD, mm^3^	2,899.7 ± 4,726.6^b^	3,201.3 ± 4,700.4
Number of Gd+ lesions, mean ± SD	1.9 ± 5.5^b^	2.0 ± 5.1

*BID, twice daily; DMF, dimethyl fumarate; EDSS, Expanded Disability Status Scale; Gd+, gadolinium-enhancing; MS, multiple sclerosis; PBO, placebo*.

a *n = 313*.

b *n = 312*.

### Brain Parenchymal Fraction

**Figure 1:**
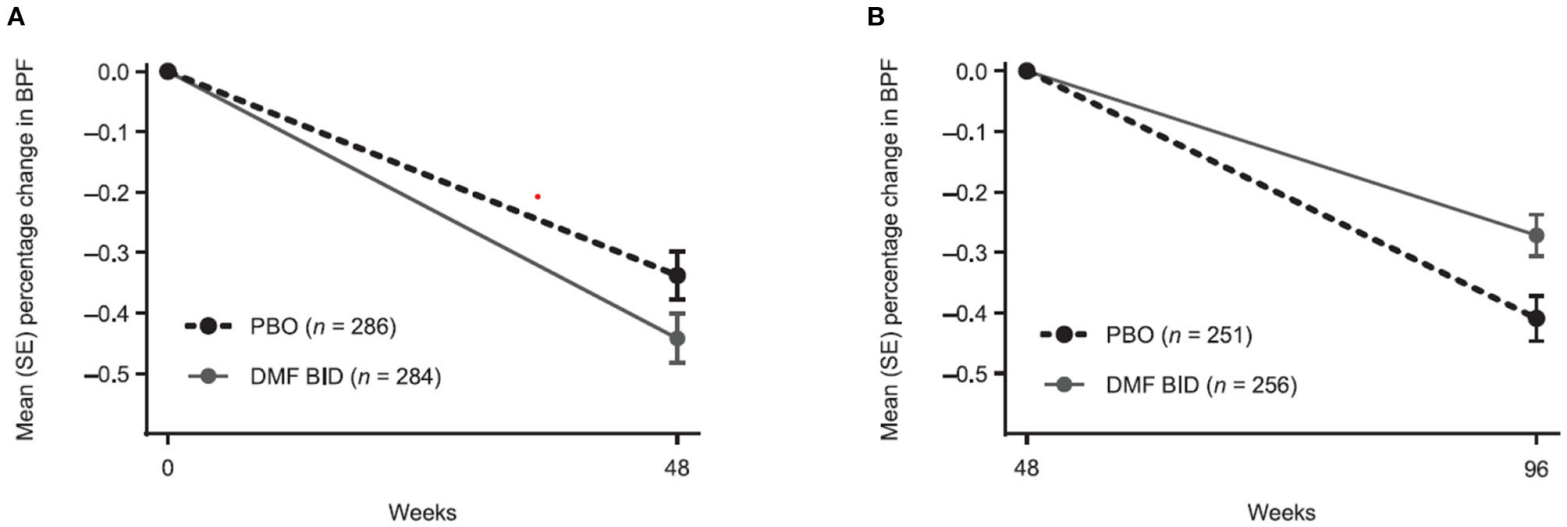
Percentage change in brain parenchymal fraction (BPF) from **(A)** baseline to week 48 and **(B)** week 48 to 96 in patients with BPF measurements at baseline and week 48, and at week 48 and week 96, respectively. BID twice daily; DMF, dimethyl fumarate; PBO, placebo.

The percentage change in BPF was analyzed from weeks 0 to 48 and weeks 48 to 96 to enable separate assessment of pseudoatrophy in the first year of treatment. From baseline to week 48, the mean (± SE) percentage change in BPF was more pronounced in the DMF BID group compared with PBO (−0.44 ± 0.04 vs. −0.34 ± 0.04) (Figure 1A). In contrast, the mean (± SE) percentage change in BPF from weeks 48 to 96 was less pronounced in the DMF BID group compared with PBO (−0.27 ± 0.03 vs. −0.41 ± 0.04) (Figure 1B). This difference represented a relative reduction in brain atrophy of 34% in the second year of DMF treatment compared with PBO.

**Figure 2:**
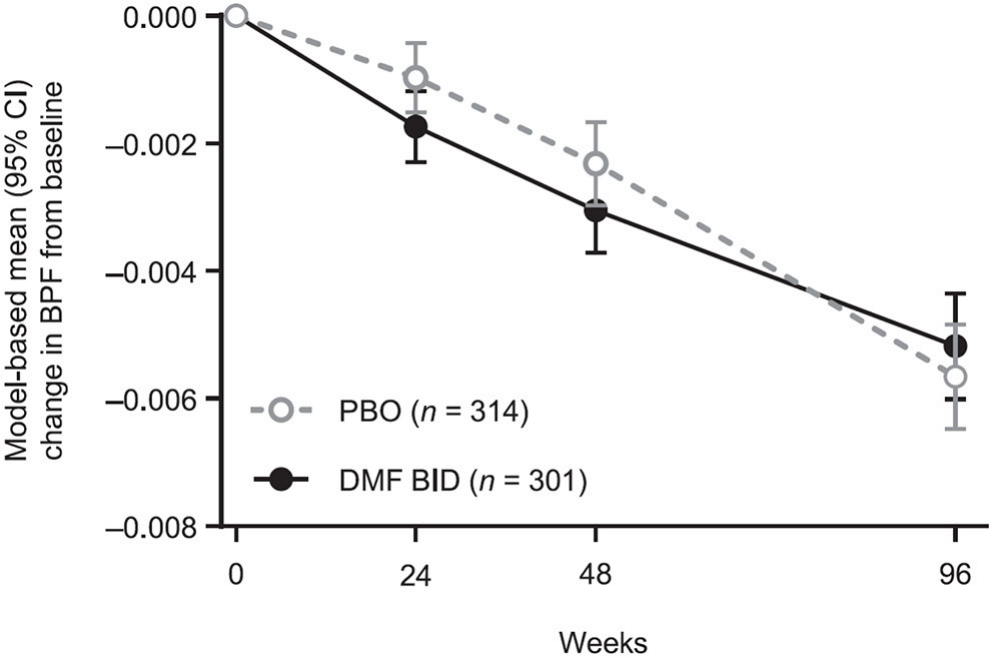
Model-based mean BPF changes from baseline, showing temporal patterns of brain volume changes by treatment in the pooled study populations. Results are obtained from a repeated measures model for change from baseline in BPF. The model includes treatment, study, week, and their two-way and three-way interactions, and is adjusted for the following covariates: region, BPF at baseline, and prior multiple sclerosis treatment (yes, no). The model has unstructured variance-covariance structure. BPF, brain parenchymal fraction; BID, twice daily; DMF, dimethyl fumarate; PBO, placebo.

The mixed-effect model for repeated measures analysis showed consistent results (Figure 2). From baseline to week 48, the least-squares mean (95% CI) change in BPF was −0.0031 (−0.0037, −0.0024) in the DMF BID group vs. −0.0023 (−0.0030, −0.0017) in the PBO group, whereas in weeks 48–96 the estimated change in BPF was −0.0021 (−0.0027, −0.0016) in the DMF group compared with −0.0033 (−0.0039, −0.0028) in the PBO group. Thus, in the second year of the study, there was a 35.9% reduction in brain volume loss for DMF BID vs. PBO (*p* = 0.0025).

### Sensitivity Analysis

A sensitivity analysis was conducted, in which observations after switching to an alternative MS medication were excluded; 17 patients in the DMF group and 42 patients in the PBO group had observations after an alternative MS medication. Compared with the primary analysis, the sensitivity analysis showed a broadly similar pattern of brain volume changes. A statistically significant adjusted mean difference in BPF change between DMF BID and PBO was observed between week 48 and 96 (0.0010 [95% CI, 0.0001, 0.0018]; *p* < 0.05).

### Correlational Analyses

**Figure 3:**
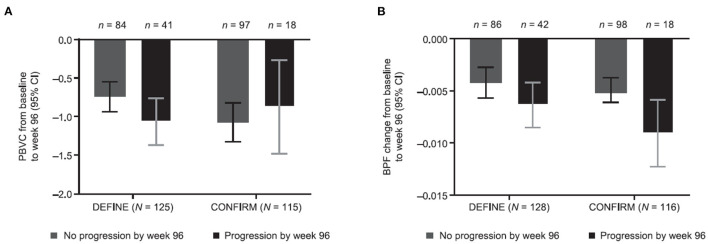
Brain volume changes stratified by disability progression and study using **(A)** PBVC from original analyses and **(B)** BPF. Original PBVC values shown are model-based estimates, and 95% CIs are based on the mixed-effect model for repeated measures for baseline to week 96 in patients with and without disability progression in the DEFINE and CONFIRM placebo groups. PBVC, percentage brain volume change; BPF, brain parenchymal fraction.

Percentage change in BPF was significantly correlated with the original PBVC measurements (Table 2). Assessing brain volume change using PBVC from the original analyses and BPF, patients with confirmed disability progression trended toward greater brain volume loss than stable patients (Figure 3). In both the PBO and DMF BID groups, mean brain volume changes from baseline to week 48 were higher in the subgroups of patients with Gd+ lesions at baseline compared with those without Gd+ lesions at baseline (Table 3). In the PBO group, these differences were persistent over time, whereas in the DMF BID group, the differences were not significant in the week 48 to week 96 timeframe.

**Table 2:** Correlations between percentage change from baseline in brain parenchymal fraction and percentage brain volume change from baseline for each study time point, by study.

**Time point**	**Spearman correlation coefficient** ^**a**^	
	**DEFINE**	**CONFIRM**
Week 24	0.601 (*n* = 255)	0.361 (*n* = 232)
Week 48	0.597 (*n* = 243)	0.523 (*n* = 228)
Week 96	0.533 (*n* = 223)	0.483 (*n* = 212)

a *All p-values < 0.0001*.

**Table 3:** Model-estimated mean brain volume changes in patients with and without baseline Gd+ lesions.

**Placebo**	**Baseline Gd+** **lesions**		**No baseline Gd+** **lesions**	
	**Placebo *n* = 138**	**DMF BID *n* = 123**	**Placebo *n* = 174**	**DMF BID *n* = 178**
Mean (95% CI) BPF change, baseline to week 48	−0.00377 (−0.00471 to −0.00282)	−0.00452 (−0.00551 to −0.00353)	−0.00114 (−0.00198 to −0.00031)	−0.00198 (−0.00281 to −0.00115)
Mean (95% CI) BPF change, week 48 to week 96	−0.00441 (−0.00524 to −0.00357)	−0.00260 (−0.00346 to −0.00175)	−0.00253 (−0.00325 to −0.00181)	−0.00182 (−0.00252 to −0.00112)

*BID, twice daily; BPF, brain parenchymal fraction; DMF, dimethyl fumarate; Gd+, gadolinium-enhancing*.

## Discussion

In a pooled analysis of brain volume loss in the DEFINE and CONFIRM phase 3 trials of DMF, a treatment effect on brain atrophy was observed with DMF compared with PBO in the second year of treatment. Results of the sensitivity analysis, which accounted for alternative medication, were similar to the primary analysis. In the first year of the study, there was no treatment effect on brain volume loss. This observation, termed the pseudoatrophy effect, is consistent with the brain volume loss patterns in several other trials, including those for IFN (13, 14), natalizumab (27), and glatiramer acetate (28).

In the 2-year trial of IFN-β1α vs. PBO in RRMS, the rate of whole brain atrophy showed a similar percentage change in BPF between IFN-β1α and PBO during the first year of treatment (*p* = 0.71) and a reduced rate of change with IFN-β1α compared with PBO during the second year of treatment (*p* = 0.03) (14). In contrast, in the pivotal AFFIRM trial, an increased rate of brain volume loss was demonstrated with natalizumab compared with PBO in the first year of therapy, with a reduction in the progression of brain atrophy compared with PBO in year 2 (29). Similarly, a *post hoc* analysis of the phase 3 CLARITY study found a pronounced pseudoatrophy effect within the first 6 months after cladribine therapy initiation. However, a statistically significant reduction in brain atrophy was reported in patients receiving short courses of cladribine compared with PBO over 2 years, and patients with lower rates of brain atrophy had the highest probability of remaining free from disability progression at 2 years (12).

In a systematic review and meta-analysis of studies of patients with RRMS reporting data on brain volume measurements, change from baseline in mean PBVC was significantly lower with disease-modifying drugs than with PBO (standardized mean difference: −0.19 [95% CI, −0.27, −0.11]; *p* < 0.001) (30). In the initial analysis of the European/Canadian glatiramer acetate vs. PBO trial, there were no differences in brain atrophy (31); however, when the MRIs were reanalyzed using SIENA, there was a difference observed in the second year of the trial (28). In addition to demonstrating pseudoatrophy, this study joins the phase 3 TEMSO study (10) in highlighting the importance of the image analysis technique used to measure brain volume loss.

Pseudoatrophy effects, evident with several disease-modifying agents, likely reflect the reduction in inflammation and resolution of associated edema following initiation of therapy. The corresponding reductions in disease activity observed during the first year of treatment in DEFINE and CONFIRM, including significant reductions in Gd+ lesions and new or enlarging T2-hyperintense lesions, lend further support to this hypothesis (17, 18, 30). In the present analyses, we found that the initial brain volume loss was more pronounced in the subgroup with baseline Gd+ lesions, signaling active inflammation at the time of treatment initiation, and that this initial (baseline to week 48) volume loss was highest in the DMF BID–treated Gd+ subgroup. This finding further supports the explanation that pseudoatrophy is the most likely explanation for the lack of treatment effect on atrophy observed in the first year.

Pseudoatrophy effects have not been noted with all disease-modifying therapies (e.g., fingolimod or teriflunomide). It is unknown why some disease-modifying therapies with anti-inflammatory effects demonstrate pseudoatrophy while others do not. It is possible that pseudoatrophy effects are more prominent when PD/T2-weighted images are used for brain volume calculations as compared with using T1-weighted images as input, possibly because image contrast in PD/T2-weighted images may be more sensitive to changes in tissue water content. Inherent differences in image analysis algorithms may also exaggerate pseudoatrophy effects in some studies as compared with others. A systematic study investigating the role of image acquisition and analysis techniques in detection of pseudoatrophy is needed to gain further understanding on this open question.

Due to a strong dependence on measurement technique, brain atrophy results should not be directly compared across studies without careful consideration and consistency across methods. BPF and PBVC are based on two fundamentally different approaches to estimate changes in brain volume over time. BPF is derived from a cross-sectional segmentation-based method, whereas SIENA is a longitudinal registration-based method; the BPF technique uses PD/T2-weighted images as input, whereas SIENA uses T1-weighted images as input. Although the overall results are consistent across techniques, confirming the treatment effects in year 2, the strengths of the correlations between PBVC and BPF and associations between brain volume loss and disability progression varied across studies. These differences highlight the fact that brain volume results from different implementations of SIENA at different MRI reading centers cannot be simply combined for pooled analyses.

The results of this analysis are strengthened by the prospective, randomized, multicenter design of the original studies and the fact that the MRI scans were analyzed by a single MRI reading center. However, the analysis was limited by the fact that neither DEFINE nor CONFIRM were prospectively planned to be powered to detect a treatment effect on brain atrophy (32). To counteract this, patient data from the DEFINE and CONFIRM studies were pooled to provide greater power for this analysis. Furthermore, the duration of this evaluation was limited to 2 years from randomization; longer-term studies have demonstrated the durable effect of DMF on whole brain atrophy (33).

To confirm the original results and enable pooled analysis of DEFINE and CONFIRM in this study, the MRIs were reanalyzed by a single reading center using the BPF approach. As with several other disease-modifying therapies, an initial reduction in brain volume was observed during the first year of DMF treatment, most likely due to a reduction in edema associated with inflammation, or pseudoatrophy effect. During the second year of treatment, DMF significantly slowed the rate of brain atrophy progression (by approximately 35%) compared with PBO. The consistency of the pooled BPF analysis with the original atrophy analyses and the observed associations with disability lend further support to the effects of DMF on brain atrophy.

## Data Availability Statement

DEFINE and CONFIRM were registered with ClinicalTrials.gov (NCT00420212 and NCT00451451). Requests for data supporting this article should be submitted to the Biogen Clinical Data Request Portal (biogenclinicaldatarequest.com).

## Ethics Statement

The studies were approved by the Relevant Institutional Review Board for each study site, and each study was conducted in accordance with the Declaration of Helsinki, International Conference on Harmonization Good Clinical Practice guidelines, and all applicable laws and regulations. All participants provided written informed consent before study procedures.

## Author Contributions

KN, CM, and EF contributed to the study design, data acquisition, analysis, interpretation of the data, and critically reviewed and revised the manuscript. OM contributed to the design of the data analysis, data analysis, interpretation, and critically reviewed and revised the manuscript. DLA, TAY, LK, and NR advised on the study design, contributed to the data acquisition and interpretation of the data, and critically reviewed and revised the manuscript. KA-R contributed interpretation of the data and drafted the manuscript. All authors provided approval of the final manuscript for submission.

## Funding

This study was funded by Biogen. Biogen was involved in the study design, data collection, data analysis, and preparation of the manuscript.

## Conflict of Interest

KN reports licensing fees from Biogen and research support from Biogen, Novartis, and Sanofi-Genzyme. OM, CM, NR, and EF were employed by Biogen. OM, CM, and EF hold stock/stock options in Biogen. DLA reports honoraria/revenue from Adelphi, Biogen, Genentech, Genzyme, MedDay, Novartis, Pfizer, Receptos, Roche, and Sanofi-Aventis; and research grant support from Biogen and Novartis. TAY reports honoraria/travel funding from Biogen, Bayer Schering, and Novartis, and research grant support from Biogen, GlaxoSmithKline, Novartis, and Schering. LK reports the following, which were received in the last 3 years and used exclusively for research support at University Hospital Basel: steering committee, advisory board, and consultancy fees from Actelion/Janssen, Bayer, Biogen, Celgene/Receptos, Genzyme, Japan Tobacco, Merck, Minoryx, Novartis, Roche, Sanofi-Aventis, and Santhera; and license fees for Neurostatus-UHB products. The Research of the MS Center in Basel has been supported by grants from Biogen, the European Union, Innosuisse, Novartis, Roche research foundations, Swiss MS Society, and Swiss National Research Foundation. NR is an employee/consultant for NeuroRx Research and a consultant for BMS, Roche, and Sanofi. KA-R is an employee of and holds stock/options in Envision Pharma Group.

## Publisher's Note

All claims expressed in this article are solely those of the authors and do not necessarily represent those of their affiliated organizations, or those of the publisher, the editors and the reviewers. Any product that may be evaluated in this article, or claim that may be made by its manufacturer, is not guaranteed or endorsed by the publisher.

## Abbreviations

BID: twice daily
BPF: brain parenchymal fraction
DMF: dimethyl fumarate
EDSS: Expanded Disability Status Scale
Gd+: gadolinium-enhancing
IFN: interferon
MS: multiple sclerosis
PBO: placebo
PBVC: percentage brain volume change
PD: proton density
RRMS: relapsing-remitting multiple sclerosis.
